# A phase I and pharmacological study of the matrix metalloproteinase inhibitor BB-3644 in patients with solid tumours

**DOI:** 10.1038/sj.bjc.6601594

**Published:** 2004-02-17

**Authors:** L Wall, D C Talbot, P Bradbury, D I Jodrell

**Affiliations:** 1 Cancer Research UK Oncology Unit, Western General Hospital, Edinburgh EH4 2XR, Scotland, UK; 2 Cancer Research UK Medical Oncology Unit, Churchill Hospital, Oxford OX3 7LJ, UK

**Keywords:** BB-3644, matrix metalloprotease inhibitor, phase I, pharmacokinetics

## Abstract

BB-3644 is an oral, broad-spectrum matrix metalloproteinase inhibitor (MMPI) structurally related to marimastat and BB-94. It is also >10-fold more active than marimastat in inhibiting the processing of cell-bound TNF-*α*. Preclinical studies suggested a favourable toxicity profile when compared to marimastat, and therefore it was selected for clinical evaluation. Patients with advanced solid tumours against which established treatments had failed, or for which no satisfactory treatment exists and of good performance status, were eligible. Treatment consisted of twice daily (bd) oral BB-3644 for 84 days. The initial dose was 5 mg bd, and subsequent cohorts were treated with 10, 20 and 30 mg bd. In all, 22 patients were enrolled. The dose-limiting toxicity (DLT) was musculoskeletal pain. For 28 days of treatment with BB-3644, 20 mg bd was the maximum tolerated dose (MTD), as at 30 mg bd, six of nine patients developed significant musculoskeletal toxicity by day 28. Following chronic oral dosing (>28 days) with BB-3644, three of five patients treated at 10 mg bd developed musculoskeletal DLT by day 84, defining the MTD as 5 mg bd. As dose-limiting musculoskeletal toxicity was encountered at doses of BB-3644 unlikely to provide an advantage over currently available MMPIs, further evaluation is not recommended.

There are more than 20 members of the matrix metalloproteinase (MMP) family of zinc-dependent proteinases, which are involved in degradation of the extracellular matrix. They have been implicated in the processes of tumour growth, invasion and metastasis (Nelson *et al*, 2000). Inhibition of the MMPs inhibits tumour cell invasion *in vitro*, and reduces metastasis formation after injection of malignant cells in xenograft models (Nelson *et al*, 2000).

The role of the MMPs in tumour invasion and metastasis prompted the development of therapeutic strategies targeting MMPs. Marimastat was the first orally bioavailable MMP inhibitor (MMPI) to enter clinical testing. Musculoskeletal pain and inflammation were the treatment-limiting toxicities (Wojtowicz-Praga *et al*, 1998; Tierney *et al*, 1999; Evans *et al*, 2001). Cumulative toxicity often necessitated treatment interruption and subsequent dose reduction or termination of therapy. Phase I and II studies reported a reduction in the rate of rise of tumour markers associated with MMPI administration, and an apparent associated prolongation of survival (Nemunaitis *et al*, 1998). Although some authors have reported clinical benefit in association with MMPI administration with respect to the reaccumulation of effusions (Macaulay *et al*, 1999) or pain (Evans *et al*, 2001), randomised studies have shown no evidence of a survival advantage following MMPI administration (Phuphanich *et al*, 2001; Bramhall *et al*, 2002a, 2002b; Shepherd *et al*, 2002; Rosenbaum *et al*, 2003).

Due to the role of MMPs in tumour invasion and metastasis, there is interest in chronic administration for patients with minimal residual disease. Attempts have therefore continued to develop MMPIs with a more favourable toxicity profile than currently available agents.

BB-3644 was developed as an oral, broad-spectrum MMPI. It is structurally related to marimastat and BB-94, in that it is a hydroxamic acid-based metalloproteinase inhibitor. It demonstrates activity against the different subtypes of MMP ranging between an IC_50_ of 3 nM against collagenase-3 to 80 nM against gelatinase A. It also shows activity against the unrelated metalloproteinase enkephalinase (IC_50_ 40 nM). It is more than 10-fold more active than marimastat in the inhibition of the processing of cell-bound TNF-*α*.

In animal studies, BB-3644 showed activity in inhibiting tumour growth in a range of tumour models including the MDA-435 human breast carcinoma and B16-BL6 murine melanoma models (British Biotech, unpublished data). In a model of lung colonisation by HODP.IP rat mammary carcinoma cells, BB-3644 had similar antitumour activity to marimastat, but, unlike marimastat, did not cause tendinitis of the hind limbs (British Biotech, unpublished data).

Toxicity studies in animals have shown the principal toxicity of BB-3644 in marmosets and rhesus macaques was cell debris in the gall bladder, with mucosal erosions and epithelial hyperplasia. In dogs, ocular changes consisting of conjunctival hyperaemia, chemosis, corneal stromal limbal vascularisation and diffuse corneal haze and flocculation were noted. Encephalopathy occurred at higher doses. In marmosets, BB-3644 induced inflammation of joint ligaments and tendons (British Biotech, unpublished data).

The primary aims of this study were:

- to determine the DLT and maximum tolerated dose (MTD) of BB-3644 administered on a protracted daily oral dosing schedule;
- to recommend a dose for further activity studies;
- to evaluate the PK parameters of BB-3644.

The secondary aim of this study was:

- To seek preliminary evidence of antitumour activity and clinical benefit.

## MATERIALS AND METHODS

### Study centres

The study was performed at the Cancer Research UK Oncology Units at the Churchill Hospital, Oxford, England and the Western General Hospital, Edinburgh, Scotland. The trial was designed to comply with the ethical principals of Good Clinical Practice in accordance with the Declaration of Helsinki. The study was approved by the Medicine and Clinical Oncology Research Ethics Subcommittee, Lothian Research Ethics Committee (Reference 1999/4/119) and the Oxford Research Ethics Committee (C00.149). All patients gave written, informed consent prior to study-screening procedures.

### Pretreatment evaluation

Patients were eligible for the study if they had a histologically proven diagnosis of a solid tumour for which no satisfactory treatment exists or against which established treatments had failed. Patients over the age of 18 were required to be of ECOG performance status 0, 1 or 2, and to have a predicted survival of at least 3 months.

All patients had satisfactory haematological function, as defined by a haemoglobin level ⩾10 g dl^−1^, neutrophil count ⩾1.5 × 10^9^ l^−1^ and a platelet count ⩾100 × 10^9^ l^−1^. They also had to have satisfactory renal and hepatic function, with a serum creatinine within the normal range (⩽110 *μ*mol l^−1^) and/or calculated creatinine clearance ⩾60 ml min^−1^, serum bilirubin ⩽17 mmol l^−1^ and other liver function tests less than twice the upper limit of the normal.

Patients with upper gastrointestinal cancers were excluded, as it was felt that this may alter the pharmacokinetics of an orally administered drug. Patients with recent ocular surgery were also excluded. In premenopausal women, pregnancy was excluded and adequate contraception was required for the duration of treatment and follow-up.

### Study design and treatment

This was a phase I, open-label, dose-escalation study. BB-3644 was supplied as capsules comprising 5, 10, 20 and 40 mg BB-3644 (with maize starch, colloidal silica, magnesium stearate and lactose) by British Biotech Pharmaceuticals Ltd, UK. BB-3644 was administered orally twice daily for 84 days to cohorts of patients at doses 5, 10, 20 and 30 mg. Dose escalation was performed in subsequent cohorts if three patients had been treated at a dose level without dose-limiting toxicity (DLT) by day 28. Dose-limiting toxicity was defined as ‘an adverse reaction likely to be caused by the study drug and unlikely to be attributed to concurrent disease or other drugs or chemicals’. Essentially, any toxicity graded by CTC criteria as at least grade II was considered to be a DLT, as this could preclude chronic oral administration of a drug. If one patient experienced DLT, the cohort size was increased to six patients for that dose level. Further dose escalation was performed, provided that no more than two of the six patients experienced DLT by day 28. Nine patients were to be treated at the highest dose level. Patients experiencing DLT were eligible to continue treatment at a reduced dose following resolution of toxicity.

### Pharmacokinetics

Blood samples were collected in heparinised tubes before treatment, every 15 min for 2 h, at 2 h 30 min, 3, 4, 6, 8, 10 and 24 h on days 1 and 14 for the measurement of BB-3644. Blood samples were kept on ice. They were centrifuged at 4°C (1500 **g** for 10 min) within 30 min of collection. The supernatant plasma was collected and frozen at −70°C or cooler, until sent for analysis. Samples were analysed centrally in the Department of Drug Metabolism and Pharmacokinetics at British Biotech, UK.

Samples were analysed for BB-3644 content using a validated reverse-phase HPLC method with mass spectrometric detection. The limit of quantification was 1 ng ml^−1^. The data were analysed using WinNonlin v2.1 Standard version (Pharsight Corporation, USA). PK parameters were derived from individual plasma concentration–time data, using noncompartmental analysis.

### Monitoring

Physical examination, chest radiograph, haematology and serum chemistry were checked at baseline, every 4 weeks and at study termination. Abdominal ultrasound scans were performed at the same times, with particular attention to the gallbladder in view of the gallbladder lesions found in animal studies. Patients were also reviewed by an opthalmologist at these times, to check the visual acuity, visual fields, and examine the whole eye and the fundi.

Disease assessment was performed at baseline and repeated at 84 days or at withdrawal from the study. Patients were reviewed 4 weeks after withdrawal from the study.

## RESULTS

### Patient demographics

Between November 1999 and March 2001, 22 patients were treated within the study. One patient entered did not fully meet the inclusion criteria on the basis of hepatic function, as alkaline phosphatase and gamma-GT were increased to twice the upper limit of the reference range, but data from this patient have been included in the analysis. Two patients had creatinine levels above that specified in the protocol, but within the normal range of their local laboratory, and were deemed eligible.

The characteristics of patients within the study are listed in Table 1

**Table 1:** Patient characteristics

Total patients treated	22
Male/female	13/9
	
*Age (years)*	
Median	55.5
Range	31–71
	
*WHO performance status*	
0	6
1	15
2	1
	
*Primary tumour site*	
Carcinoma (not otherwise specified)	5
Cervical cancer	2
Colorectal cancer	6
Lung cancer	8
Ovarian cancer	1
	
*Previous treatment*	
Surgery	13
Chemotherapy	19
Radiotherapy	12
Hormone/immuno/biological therapy	2

. The patients were relatively young (median age 55.5 years, range 31–71 years). The majority of patients (86%) had previously received chemotherapy.

### Treatment administered

Early dose levels were expanded, as per protocol, due to patient drop-out before day 28. At 5 mg bd, two patients withdrew within 4 weeks, one due to an adverse event not considered to be related to the study drug, and one due to disease progression. These patients were replaced in the study. As these events were not considered to be related to the study drug, this was not considered to represent DLT. At 10 mg bd, again patient withdrawal was not considered to be related to the study drug, so dose escalation continued. At 20 mg bd, all patients completed 28 days of treatment without experiencing DLT, so the dose was increased to 30 mg bd. At this dose, two patients received less than 4 weeks of treatment, and so were replaced. Three other patients were withdrawn before completion of the 84 days of treatment, two due to adverse events and one due to disease progression. For 28 days of treatment with the drug 20 mg bd was therefore considered to be the MTD.

One patient, treated at 10 mg bd, had stable disease after 84 days of treatment, and continued the study drug on an extension protocol. While in the extension phase of the study, she had a pulmonary embolus, and subsequently further thromboembolic episodes despite adequate anticoagulation. She died after 250 days on study, after a probable cerebrovascular infarction with a left-sided hemiparesis.

One patient in the 30 mg bd group received 20 mg on days 0 and 1, and so, for PK analysis, was included with the 20 mg bd group on day 0 and with the 30 mg bd group on day 14.

### Adverse events and DLTs

There was no significant opthalmological or gall bladder-related toxicity seen. All patients did, however, experience adverse events during the study. These were considered to be treatment-related in three patients at 5 mg bd, five at 10 mg bd, one at 20 mg bd and eight at 30 mg bd. The most common treatment-related events were musculoskeletal in nature and were reported for one, three, one and eight patients, respectively. Recovery from musculoskeletal toxicity was often delayed, but was not related to the dose, type of event or NCI CTC grade. At 5–20 mg bd, 94% of musculoskeletal events resolved, with a median time to resolution of 175 days. At 30 mg bd, only 78% of events resolved, but the median time to resolution was 49 days.

The musculoskeletal events were considered dose-limiting in three patients at 10 mg bd, and six at 30 mg bd (Table 2

**Table 2:** Dose-limiting toxicity

	**Toxicity within 28 days**	**Toxicity at any time**
5 mg bd	1/5 (20%)	1/5 (20.0%)
10 mg bd	0/5	3/5 (60.0%)
20 mg bd	0/3	1/3 (33.3%)
30 mg bd	4/9 (44%)	6/9 (66.7%)

). The dose was escalated, according to the protocol, if three patients had been treated at that dose for 28 days, without experiencing DLT. The dose was therefore increased, despite the high proportion of patients experiencing DLTs at early dose levels.

Two patients experienced dose-limiting malaise (NCI CTC toxicity >grade 1) on treatment, considered to be possibly related to the study drug. Two patients developed phlebitis, and one of these patients subsequently had a deep venous thrombosis and pulmonary embolism. Again, there was considered to be a possible relationship to the study drug. Other symptoms considered to be possibly related to the study medication were taste changes (one patient), shortness of breath (one patient), neurosensory changes (one patient) and anorexia (one patient).

Serious adverse events (SAEs) consisted of events requiring hospitalisation (seven patients) and events resulting in death (two patients). SAEs were reported for eight patients during the main study and for a ninth patient during the continuation of treatment.

One SAE was considered to be possibly related to the study drug (thromboembolic episodes). This was attributed to the drug, on the basis of the temporal relationship between drug administration and the development of thromboembolic phenomena. Thromboembolic episodes have not previously been reported in relation to MMPI administration.

All other SAEs were considered to be related to the underlying malignancy. These consisted of sepsis (one patient); nausea and vomiting (two patients); hypercalcaemia (two patients); abdominal pain (two patients) and acute renal failure (one patient).

### Assessment of response

Response was formally assessed radiologically after 3 months of therapy. Of the 13 patients that completed 12 weeks of therapy, two had stable disease and 11 had progressive disease radiologically. Three patients completed less than 28 days of therapy, and disease was therefore deemed nonevaluable. Five patients progressed clinically and one patient progressed radiologically prior to the 3-month timepoint. It was therefore concluded that in our study we had no evidence of beneficial therapeutic activity.

### Plasma pharmacokinetics

**Figure 1:**
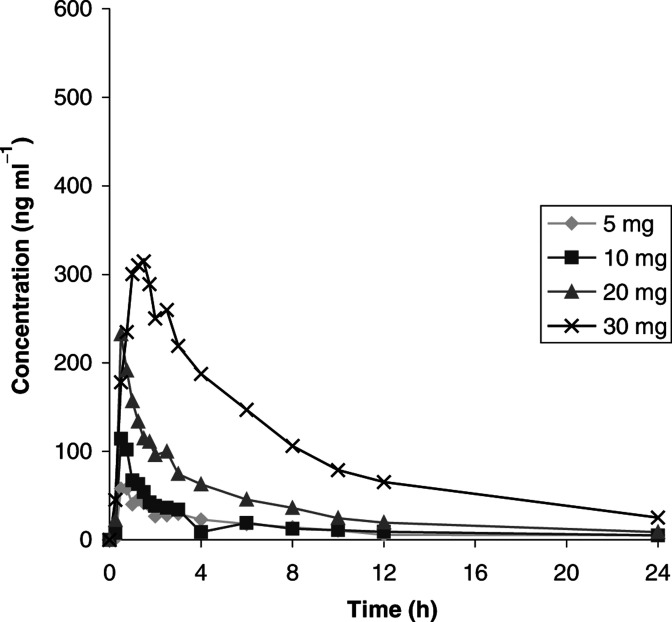
Mean plasma concentration of BB-3644 on day 0.

On the days of PK sampling, only the morning dose of BB-3644 was taken. On day 0, BB-3644 was rapidly absorbed (Figure 1

; Table 3

**Table 3:** Mean PK parameters following a single oral dose of BB-3644

	**5 mg bd**		**10 mg bd**		**20 mg bd**		**30 mg bd**	
	**Day 0**	**Day 14**	**Day 0**	**Day 14**	**Day 0**	**Day 14**	**Day 0**	**Day 14**
C_max_ (ng ml^−1^)	58	134	114	179	232	282	315	536
*T*_max_ (min)	45	60	30	60	30	45	90	105
*T*_1/2_ (min)	120	240	90	150	90	180	360	360
AUC_0–24_ (ng h ml^−1^)	331	903	388	989	781	1248	2523	4401

Patient 18 received 20 mg bd in error on days 0 and 1, but his data are included with those of the 30 mg bd group.

), achieving a mean maximal concentration between 30 and 90 min following administration. The maximal concentration achieved was dose-dependent and linear, varying between 58 and 315 ng ml^−1^ for the maximum BB-3644 dose. The initial decline in plasma concentrations was rapid and elimination was multiphasic, with BB-3644 detectable 24 h after dosing.

**Figure 2:**
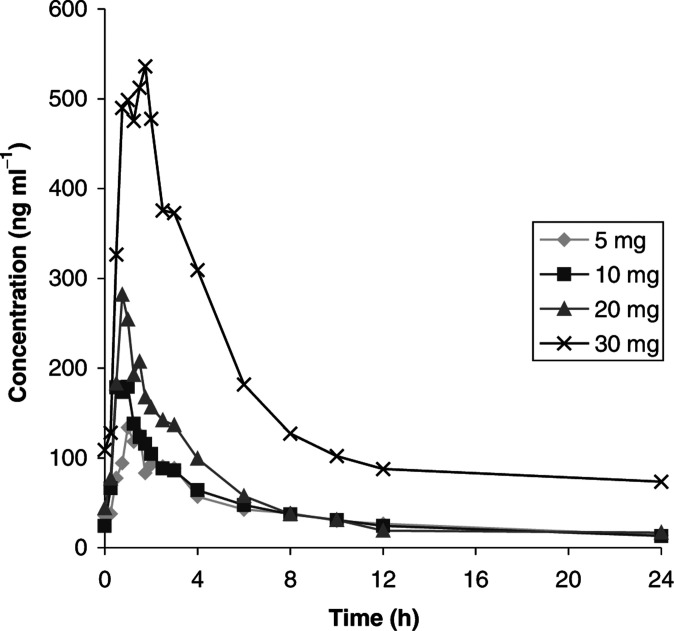
Mean plasma concentration of BB-3644 on day 14.

After repeated oral administration (day 14) (Figure 2

and Table 3), the drug was again rapidly absorbed. The mean maximum concentration achieved was higher than that on day 1 (134–536 ng ml^−1^).

## DISCUSSION

In this phase I study of the novel matrix metalloprotease inhibitor BB-3644 administered by mouth twice daily, the start dose was 5 mg bd and the dose was escalated to a dose of 30 mg bd, based on the incidence of toxicities before day 28. Dose escalation was discontinued at a dose of 30 mg bd, due to the development of significant musculoskeletal toxicity in six out of nine patients by day 28.

When treatment was continued beyond 28 days, however, patients developed DLT at ALL doses of BB-3644. For a 28-day administration of BB-3644, 20 mg might be considered to be the MTD. However, it is anticipated that administration of such agents should be tolerable over longer periods and therefore, for chronic oral administration, 5 mg bd could have been considered to be the MTD, as three out of five patients developed DLT at 10 mg bd within 84 days.

The DLT was musculoskeletal. Musculoskeletal DLTs were recorded in 14 of the patients (64%). No other DLT was observed in more than two patients (9% of patients).

PK analysis demonstrated that the BB-3644 was rapidly absorbed when orally administered and the drug was still identifiable after 24 h. At the maximum dose of BB-3644, we achieved an AUC of 4401 ng h ml^−1^. In the phase I studies of marimastat, the AUC achieved at the MTD was 2623 ng h ml^−1^ (Wojtowicz-Praga *et al*, 1998), which had been our target AUC in this study. The PK assessment confirmed that our dosing strategy had been appropriate to achieve adequate plasma exposure of the drug.

It had been hoped that, by virtue of its different biochemical profile, BB-3644 would be better tolerated than marimastat with respect to musculoskeletal toxicity. Indeed, preclinical cancer models and toxicity studies had indicated that an improved therapeutic margin might be achievable in patients. The tolerability of BB-3644 in our study was no better than that reported in phase I and II studies of marimastat, with a very similar pattern of musculoskeletal toxicity (Wojtowicz-Praga *et al*, 1998; Tierney *et al*, 1999; Evans *et al*, 2001).

In view of the toxicity that was experienced at dose levels associated with similar plasma exposure, it was felt unlikely that the drug offered any benefit over currently available MMPIs and clinical development was terminated.

## CONCLUSION

Chronic oral administration of BB-3644 in patients with cancer is associated with musculoskeletal toxicities from doses of 5 mg bd upwards, and offers no advantage over marimastat in terms of systemic exposure and tolerability.
